# Prevalence, Treatment Eligibility and Postoperative Survival for Europeans with Aortic Stenosis

**DOI:** 10.1186/1749-8090-10-S1-A162

**Published:** 2015-12-16

**Authors:** Paolo De Sciscio, Jacob Brubert, Michele De Sciscio, Marta Serrani, Joanna Stasiak, Geoff D Moggridge

**Affiliations:** 1 Department of Engineering, University of Cambridge, Trumpington Street, Cambridge CB21PZ, UK; 2 Department of Chemical Engineering & Biotechnology, University of Cambridge, Pembroke Street, Cambridge, CB23RA, UK; 3 Internal Medicine Service, Royal Adelaide Hospital, North Terrace, Adelaide 5000, Australia

## Background/Introduction

Aortic Stenosis (AS) is the most frequent valvular pathology in the developed world. Whilst much is known about its pathogenesis and treatment, a paucity of data exists on the prevalence and number of patients eligible for valve replacement.

## Aims/Objectives

To quantify prevalence, treatment eligibility and postoperative survival in patients with AS.

## Method

A systematic search was conducted across PubMed and EMBASE for studies evaluating prevalence, severity, decision-making and survival in AS patients. Studies were selected using a priori defined criteria reviewed by two independent investigators. Prevalence rates [95%CI] were calculated and pooled using fixed and random-effects models (statistical heterogeneity evaluated via X2 and I2). Subsequently, Monte Carlo methods with beta distributions and 2012 population data were utilised to assess eligibility per treatment option (using ESC indications).

## Results

Fifty-four studies were included encompassing 52,951 patients across 5 domains: prevalence, severity, symptom status, treatment and outcome. Pooled prevalence in the general population aged 55-74yrs and >75yrs was 2.9% [1.5-4.3%] and 13.6% [8.3-18.9%], respectively. Of these, 21.6% [19.1-24.2%] had severe AS with 71.1% [62.7-79.4%] symptomatic. SAVR was performed in 57.1% [47.7-66.6%] of symptomatic patients and 28.2% [16.6-39.7%] of asymptomatic patients. In non-operated asymptomatic patients, 44.7% [36.7-53.0%] progressed to SAVR within 2yrs. Regardless of symptoms, SAVR was associated with a 3-fold increase in survival within 2yrs (OR: 3.6 and 3.9). In high-risk/inoperable severe symptomatic patients aged ≤75yrs, 39.9% [31.4-48.4%] received TAVR. On Monte Carlo simulation, there are 9,187,586 [6,988,043-11,629,367] Europeans aged ≤55yrs with AS. Of these, 1,984,666 [1,494,584-2,550,594] have severe AS with 1,178,766 [878,802-1,528,812] eligible for SAVR and 234,934 [149,699-340,960] eligible for TAVR.

## Discussion/Conclusion

Within current ESC guidelines, approximately 1.2 million Europeans aged ≤55yrs are candidates for SAVR with an additional 230,000 patients aged ≤75yrs eligible for TAVR.

